# Demographic and clinical profiles of *Plasmodium falciparum* and *Plasmodium vivax* patients at a tertiary care centre in southwestern India

**DOI:** 10.1186/s12936-016-1619-5

**Published:** 2016-11-25

**Authors:** Laura Chery, Jennifer N. Maki, Anjali Mascarenhas, Jayashri T. Walke, Pooja Gawas, Anvily Almeida, Mezia Fernandes, Marina Vaz, Rakesh Ramanan, Diksha Shirodkar, Maria Bernabeu, Suresh Kumar Manoharan, Ligia Pereira, Rashmi Dash, Ambika Sharma, Riaz Basha Shaik, Rimi Chakrabarti, Prasad Babar, John White, Devaraja G. Mudeppa, Shiva Kumar, Wenyun Zuo, Kristen M. Skillman, Usheer Kanjee, Caeul Lim, Kathryn Shaw-Saliba, Ashwani Kumar, Neena Valecha, V. N. Jindal, Anar Khandeparkar, Pradeep Naik, Sunanda Amonkar, Manoj T. Duraisingh, Shripad Tuljapurkar, Joseph D. Smith, Nagesh Dubhashi, Roque G. W. Pinto, Maria Silveria, Edwin Gomes, Pradipsinh K. Rathod

**Affiliations:** 1 Departments of Chemistry and Global Health, University of Washington, Seattle, WA 98195 USA; 2 Goa Medical College and Hospital, Bambolim, Goa 403202 India; 3 Center for Infectious Disease Research (CIDR), Seattle, WA 98109 USA; 4 Department of Biology, Stanford University, Stanford, CA 94305 USA; 5 Department of Immunology and Infectious Diseases, Harvard T. H. Chan School of Public Health, Boston, MA 02115 USA; 6 National Institute of Malaria Research, Field Unit, Campal, Goa 403001 India; 7 National Institute of Malaria Research (ICMR), Sector 8, Dwarka, New Delhi 110077 India

**Keywords:** MESA-ICEMR, Goa, Epidemiology, Diagnostics, Severity, Characteristics, Features

## Abstract

**Background:**

Malaria remains an important cause of morbidity and mortality in India. Though many comprehensive studies have been carried out in Africa and Southeast Asia to characterize and examine determinants of *Plasmodium falciparum* and *Plasmodium vivax* malaria pathogenesis, fewer have been conducted in India.

**Methods:**

A prospective study of malaria-positive individuals was conducted at Goa Medical College and Hospital (GMC) from 2012 to 2015 to identify demographic, diagnostic and clinical indicators associated with *P. falciparum* and *P. vivax* infection on univariate analysis.

**Results:**

Between 2012 and 2015, 74,571 febrile individuals, 6287 (8.4%) of whom were malaria positive, presented to GMC. The total number of malaria cases at GMC increased more than two-fold over four years, with both *P. vivax* and *P. falciparum* cases present year-round. Some 1116 malaria-positive individuals (mean age = 27, 91% male), 88.2% of whom were born outside of Goa and 51% of whom were construction workers, were enroled in the study. Of 1088 confirmed malaria-positive patients, 77.0% had *P. vivax,* 21.0% had *P. falciparum* and 2.0% had mixed malaria. Patients over 40 years of age and with *P. falciparum* infection were significantly (*p* < 0.001) more likely to be hospitalised than younger and *P. vivax* patients, respectively. While approximately equal percentages of hospitalised *P. falciparum* (76.6%) and *P. vivax* (78.9%) cases presented with at least one WHO severity indicator, a greater percentage of *P. falciparum* inpatients presented with at least two (43.9%, *p* < 0.05) and at least three (29.9%, *p* < 0.01) severity features. There were six deaths among the 182 hospitalised malaria positive patients, all of whom had *P. falciparum.*

**Conclusion:**

During the four year study period at GMC, the number of malaria cases increased substantially and the greatest burden of severe disease was contributed by *P. falciparum*.

## Background

Globally, parasites of the *Plasmodium* genus infect more than 200 million people and cause an estimated 438,000 deaths annually [1]. India is the second most populous country in the world with ongoing malaria transmission, with 91% of its more than 1.2 billion population living in areas of malaria risk [1]. The most recent estimates report up to 26 million malaria cases and 55,000 deaths due to malaria annually in India [1]. However, there is ongoing, vigorous debate about these figures, in part due to the vast scale of the country [2–5].

India is co-endemic for *Plasmodium falciparum* and *Plasmodium vivax*, posing challenges for malaria control and elimination planning because the two parasite species may differ in mosquito vectors, spatial distributions and transmission dynamics and because of the relapsing nature of *P. vivax* infection with a dormant liver stage [6–8]. Overall, approximately 66% of malaria infections in India are caused by *P. falciparum* and 34% are caused by *P. vivax* [1]. However, the proportional distribution varies across India and a wide range of clinical presentations are seen from both predominant species of malaria [6]. In contrast to Africa, malaria transmission in India is more limited, both adolescents and adults are at risk of severe malaria, and a substantial proportion of cases are infected with *P. vivax* rather than the traditionally more virulent *P. falciparum*. Although many expansive and comprehensive studies have been carried out in Africa [9–14] and Southeast Asia [15–20] to examine pathogenesis and mortality determinants in malaria-positive patients, a more limited number of such studies, generally smaller in scope, have been conducted within India [21–29].

India has one of the world’s largest and most extensive national surveillance systems to identify malaria incidence [30, 31] and is a highly heterogeneous country with more than 2000 ethnic groups and 22 official languages spread across 29 states and seven union territories. All of these states have populations in the millions and a more than ten-fold variation in average per capita income [32], which leads to important geographic variations in disease epidemiology and substantial variability in the delivery of malaria diagnosis, care and treatment across the country [33]. It would be costly and operationally difficult to measure all of the diverse malaria settings in India through community-based surveys or reactive case-detection methods [34] and challenging to comprehensively address every environmental context through national-level strategies, programmes and recommendations [35, 36].

In addition to India’s vast countrywide and state-level monitoring of malaria, rigorous hospital-based reports can provide epidemiological data relevant to local control and elimination initiatives as well as clinical data relevant to diagnosis, care and treatment efforts. Clinicians, as well as the wider public health system, may benefit from deep studies of specific patient pools in order to better understand malaria transmission and pathogenesis in local communities. Such information may also assist in the prioritization of resources for patient care and treatment.

With the aim of conducting methodical studies in a low to mid-endemicity, peri-urban setting, the Malaria Evolution in South Asia (MESA) International Center of Excellence for Malaria Research (ICEMR) established a research site at Goa Medical College and Hospital (GMC) [37–39]. Previously an overseas province of Portugal, Goa is a small, prosperous, southwestern state of India where both *P. falciparum* and *P. vivax* are endemic. GMC is the only government tertiary care centre in the state and operates under the auspices of the Government of Goa Public Health Department to provide health care to all, free-of-charge. The relatively advanced diagnostic and clinical capabilities at GMC draw a large, diverse patient pool and allow for deep clinical analysis. As a research site, GMC offers a highly heterogeneous patient population, a constant flow of febrile and malaria-positive cases, and a wide spectrum of clinical presentations of malaria in a relatively affluent, burgeoning peri-urban area. The present study provides a detailed description of the demographic, diagnostic and clinical characteristics of malaria-positive study participants at GMC from 2012 to 2015.

## Methods

### Institutional ethics approvals

The present work was part of the US National Institutes of Health-sponsored Program Project [40, 41] entitled Malaria Evolution in South Asia International Center of Excellence for Malaria Research (MESA-ICEMR). The activities of this centre were approved by the Government of India (GOI) Health Ministry Screening Committee (HMSC) and the Government of Goa Public Health Department. The human subjects protocol and consent forms for enrolment of *Plasmodium*-positive individuals presenting to Goa Medical College and Hospital (Bambolim, Goa, India) were approved by the institutional review boards (IRB) of the Division of Microbiology and Infectious Diseases (DMID) at the US National Institute of Allergy and Infectious Diseases (NIAID), GMC, and the University of Washington (UW).

### Study design and enrolment

All febrile individuals presenting to the outpatient, paediatric, and casualty departments of GMC were tested by hospital staff for *Plasmodium* infection via finger-prick or venous blood draw and examination by microscopy of Giemsa-stained thin blood smear and/or by rapid diagnostic test (RDT), FalciVax, Zephyr Biomedicals). The hospital generally only used one method of testing per individual, usually microscopy. In rare instances, both RDT and microscopy were used. If the RDT or microscopy was positive, the individual was counted as malaria positive. If the RDT and microscopy showed different species of parasite, the individual was counted as mixed infection positive. Hospital-determined malaria positivity and species of infection results are presented for all febrile patients presenting to GMC between January 2012 and December 2015.

Individuals determined to be malaria positive by either microscopy or RDT by the hospital, who were between the ages of 12 months and 65 years and not pregnant, were referred to the MESA-ICEMR study team. Enrolment generally occurred during normal working hours and when study staff were not completing enrolment of a previous patient.

Outpatients and individuals admitted to the Medicine, Paediatric, and Intensive Cardiac Care Unit (ICCU) wards were approached for participation in the study. Severely ill individuals requiring use of a ventilator and, therefore, admitted to the Intensive Care Unit (ICU) were not approached for inclusion in this study. Malaria-positive individuals were given oral and written descriptions of the study and were asked to provide written informed consent or, in the case of children between 12 months and 18 years, consent of a parent or guardian or, in the case of children between the ages of eight and 18 years, assent in addition to the consent of a parent or guardian. Each study participant received a unique numerical code in order to streamline data collection.

Upon enrolment, study participants provided demographic information as well as history of malaria infection, symptoms and travel to study staff. Following venous blood draw into vacutainers (ACD, BD, India), study participants received care and treatment as directed by the attending physician at GMC.

### Sample processing

Each venous blood sample was transferred to the on-site MESA-ICEMR research laboratory. Research staff immediately prepared three thin and two thick blood smears. *Plasmodium* species and parasitaemia were determined by Giesma-stained thick smear reading by expert microscopists. Research staff then performed an additional RDT (FalciVax, Zephyr Biomedicals, India) and measured haemoglobin (Hb 201, HemoCue, USA) and haematocrit (Iris StatSpin, Beckman Coulter, USA).

In addition to the hospital determination used for initial recruitment, research study staff also made a separate, independent determination of malaria positivity and species of infection based on diagnostic tests conducted in the MESA-ICEMR laboratory. If both RDT and microscopy results were negative in the MESA-ICEMR lab, the patient was classified as an unconfirmed case. If RDT or microscopy was positive, the patient was counted as a confirmed malaria positive case. If the RDT and microscopy showed different species of parasite, the sample was identified as mixed infection positive. Individuals enroled in the study who were determined to be malaria positive by the hospital, but who were classified as malaria negative by the research study staff were excluded from statistical analysis of demographic, diagnostic and clinical characteristics. Study-determined positivity and species of infection results are presented from April 2012 through December 2015.

### Clinical characteristics and severity scores

Measured features among all enroled patients with confirmed malaria infection were: high parasitaemia (>4.0% for *P. falciparum*, >1.5% for *P. vivax*); hyperparasitaemia (>10% for *P. falciparum*, >2.0% for *P. vivax)*; high fever (>38.1 °C); severe fever (>38.9 °C); anaemia (haemoglobin (Hb) < 9 g/dL in those 12 years and older, Hb < 7 g/dL in those 1–11 years); and severe anaemia (Hb < 7 g/dL in those 12 years and older, Hb < 5 g/dL in those 1–11 years). Patients were admitted to GMC based on the clinical judgement of the attending physician and did not have to fulfil any WHO criteria for severe malaria classification.

Enroled *P. falciparum*- and *P. vivax*-positive patients who were hospitalised were assessed daily by trained GMC clinicians for severity of infection based on WHO criteria [42, 43]. Measured features among enroled, hospitalised patients associated with severe malaria infection were: cerebral malaria (Glasgow coma score <11 in adults or Blantyre coma score <3 in children and presence of asexual forms in blood); hypoglycaemia (blood glucose < 40 mg/dL); metabolic acidosis (plasma bicarbonate < 15 mmol/L), renal failure (serum creatinine > 3.0 ml/dL and/or blood urea nitrogen (BUN) > 17 mmol/L); abnormal bleeding (observable); respiratory distress (breathing rate > 20 breaths/min or partial oxygen (PaO_2_) < 75); severe jaundice (total bilirubin > 10 mg/dL); circulatory collapse/shock (systolic blood pressure (BP) < 80 mmHg with cold extremities); pulmonary oedema (observable); severe anaemia (Hb < 7 g/dL or haematocrit (HCT) < 20% in those 12 years and older, Hb < 5 g/dL or HCT < 15% in those one to 11 years); and death. Occasionally, the complete clinical laboratory investigation panel was not ordered [42, 43]. A severity score (SS) was calculated for all hospitalised patients based on the total number of WHO severe malaria criteria met at enrolment and throughout hospitalisation.

### Meteorological data

Rainfall data for Goa were obtained from the India Meteorological Department (IMD) (Ministry of Earth Sciences, Government of India). Data were collected at the IMD observatory in the capital city of Goa, Panaji, located roughly 5 km from GMC.

### Data and sample management

Demographic and clinical study data were collected and managed using REDCap electronic data capture tools (Nashville, TN, USA). Diagnostic study data were recorded and stored using LabKey software (Seattle, WA, USA). All samples and associated aliquots were labelled and stored using a customized FreezerPro database (RURO Inc, Frederick, MD, USA).

### Statistical analysis

Data were initially entered into a customized SQL database (LabKey server) followed by independent verification against the original case report forms. Statistical differences between percent parasitaemias between species for all enroled patients, inpatients and outpatients were determined using an unequal variance *t* test using GraphPad Prism 6 software (La Jolla, CA, USA). All other analyses were conducted using R (Vienna, Austria).

For univariate analysis, the primary outcome was the species of malaria infection. Continuous variables were summarized with mean and standard deviation and binary variables were summarized with proportions. Between group univariate comparisons of features for *P. falciparum* and *P. vivax* were analysed with logistic regression. Demographic and diagnostic features were reported for all confirmed *P. falciparum* and *P. vivax*-infected enroled patients. Clinical laboratory tests and classifications were only reported for confirmed *P. falciparum* and *P. vivax*-infected inpatients and were included in tables only if a threshold of 20% of enroled inpatients had a particular documented clinical result or classification. Logistic regression was also used to measure differences in hospitalisation rates by species of infection between age ranges. Results were represented as odds ratios with 95% confidence intervals (OR (95%)) as well as *p* values. Differences were considered to be significant at *p* values ≤0.05.

Mixed *P. falciparum* and *P. vivax* infections were excluded from all statistical analyses due to the small sample size.

## Results

### Febrile and malaria-positive cases at Goa Medical College and Hospital

**Fig. 1:**
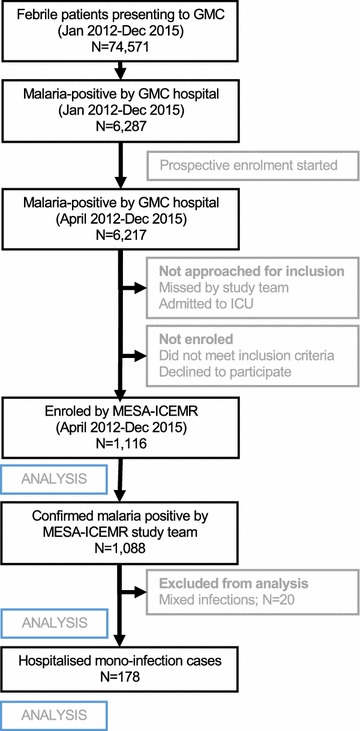
Diagram of study enrolment at Goa Medical College and Hospital. Of the 74,517 febrile patients who presented to GMC and were tested for malaria, 6287 were positive by hospital diagnosis. Of the 6287 cases identified by the hospital between January 2012 and December 2015, 6217 were referred to the MESA-ICEMR study team between April 2012 and December 2015. Of 6217, 1116 study participants were enroled by the MESA-ICEMR and 1088 were confirmed by the MESA-ICEMR to be malaria positive. Excluding mixed infections, 178 confirmed malaria cases were hospitalised by GMC

**Fig. 2:**
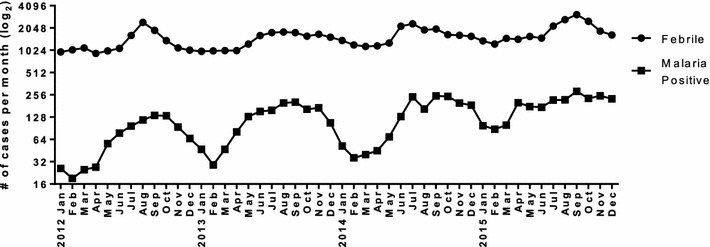
Increasing febrile and malaria positive cases at Goa Medical College and Hospital over four years. In 2012, 889 malaria cases were identified out of 15,589 febrile cases. In 2013, 1477 malaria cases were identified out of 17,017 febrile cases. In 2014, 1660 malaria cases were identified out of 19,454 febrile cases. In 2015, 2261 malaria cases were identified out of 22,511 febrile cases

**Fig. 3:**
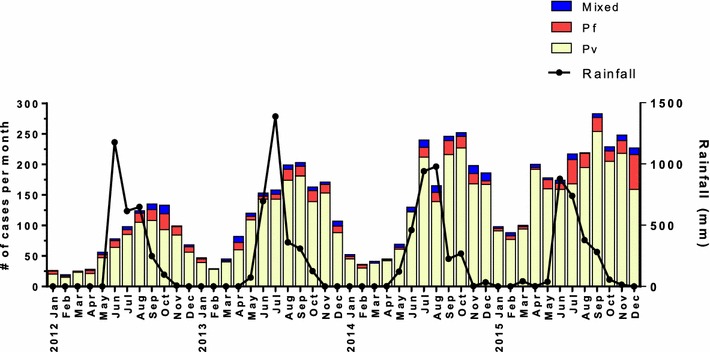
Monthly malaria positive cases by species with rainfall data over four years. In 2012, 721 *P. vivax*, 120 *P. falciparum* and 48 mixed malaria cases were diagnosed for a total of 889 annual malaria infections at GMC. In 2013, 1297 *P. vivax*, 116 *P. falciparum* and 64 mixed malaria cases were diagnosed for a total of 1477 annual malaria infections at GMC. In 2014, 1467 *P. vivax*, 111 *P. falciparum* and 82 mixed malaria cases were diagnosed for a total of 1660 annual malaria infections at GMC. In 2015, 1972 *P. vivax*, 223 *P. falciparum* and 66 mixed malaria cases were diagnosed for a total of 2261 annual malaria infections at GMC

A total of 74,571 febrile individuals presented to GMC between January 2012 and December 2015 and all were tested for malaria. Of those, 6287 (8.4%) were determined to be positive for malaria infection (Fig. 1). Over four years of passive surveillance, the number of malaria-positive cases presenting to GMC steadily and significantly increased, from 889 cases in 2012 to 2261 cases in 2015 (Fig. 2). While the traditional malaria season in Goa is June to December, *P. falciparum* and *P. vivax* cases were recorded throughout the year at GMC with the peak coinciding with the rainy season (Fig. 3). In 2015, the increase in malaria cases preceded the annual rains and case numbers remained high from April through December. The number of monthly cases observed at GMC during the height of the malaria season (September) was approximately five times greater than at the lowest point during the middle of the dry season (February).

Of the malaria-positive individuals presenting to GMC, the majority (86.8%) were diagnosed by the hospital with *P. vivax* mono-infection. *Plasmodium falciparum* infections accounted for 9.1%, and mixed infections for 4.1% of the total cases over four years (Fig. 3). Month-wise, the number of *P. vivax* infections was always greater than *P. falciparum* and mixed infections. The seasonality of *P. vivax* and *P. falciparum* infections was very similar, with case numbers of both increasing after the start of the rains. At the peak of the malaria transmission season as many as 10% of fever cases at GMC were malaria positive (Fig. 2).

### Demographic characteristics of study participants

A total of 1116 of 6217 (18%) febrile individuals identified by GMC as malaria-positive via microscopy or RDT were enroled in the present study between April 2012 and December 2015 (Fig. 1). Enrolment of this subset of patients was based on the logistical capabilities of the study team. All study participants had a mean age of 27 years (median 24 years; IQR 20–32 years) and were predominantly male (91.0%). The male-to-female ratio of study participants was roughly similar to the gender distribution of febrile cases presenting to GMC (86.7% male and 13.3% female). The mean age of male study participants was 27 years (median 24 years, IQR 20–31), while the mean-age of non-pregnant female study participants was slightly older at 30 years (median 31 years, IQR 19–40). A small minority of malaria-positive cases (51, 4.6%) were children under the age of 16 years, of whom 64.7% were male and 35.3% were female.

**Fig. 4:**
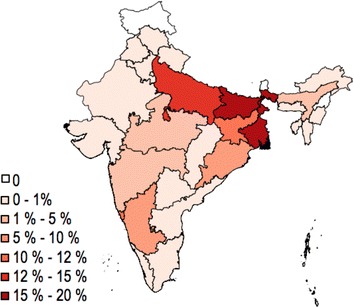
Geographic heterogeneity of Goa Medical College malaria-positive study participants. Study participants at GMC represented a very heterogeneous Indian population and most study participants were born outside of Goa. The proportion of patients that were born in each state is shown

Study participants at GMC represented a very heterogeneous Indian population. Approximately one-tenth of study participants (11.8%) were born in Goa and 88.2% were born in 26 other Indian states or outside of India (Fig. 4). The states with the greatest representation of study participants were the eastern states of Bihar with 186 (16.7%) individuals, West Bengal with 179 (16.0%), Uttar Pradesh with 152 (13.6%), and Jharkhand with 111 (9.9%). The majority of study participants (51.5%) self-identified as construction workers while 4.7% self-identified as students, 3.0% as housewives, 1.7% as soldiers or police, 1.5% as office workers, 1.3% as factory workers, and 33.2% selected the option other.

Some 97.5% of study participants were confirmed to be *Plasmodium* positive by the MESA-ICEMR study team, with 77.0% of those cases being *P. vivax*, 21.0% *P. falciparum* and 2.0% mixed-infection positive. The majority of malaria-positive individuals (83.3%) were classified as having uncomplicated malaria and treated on an outpatient basis based on the clinical judgement of the attending physician at GMC. While *P. vivax* infections were much more common than *P. falciparum*, a higher proportion of hospitalised cases were *P. falciparum* positive (107/182, 58.8%) versus *P. vivax* positive (71/182, 39.0%) and mixed-infection positive (4/182, 2.2%). All analyses presented below includes only patients with mono-infections.

### Demographic and diagnostic characteristics of confirmed malaria patients

As expected, malaria patients infected with *P. falciparum* only were significantly more likely to have a higher parasitaemia than those infected with *P. vivax* mono-infection among both outpatient and inpatient groups (Table 1). The mean parasitaemia value among *P. vivax* cases was slightly higher for outpatients as compared to inpatients, though the difference was not significant.

**Table 1:** Parasitaemia profiles by unequal variances *t* test of *Plasmodium falciparum* and *Plasmodium vivax* patients

	Number of cases^$^		Per cent parasitaemia (Mean ± SD)		*p* value
	*P. falciparum*	*P. vivax*	*P. falciparum*	*P. vivax*	
Outpatients	121	767	1.30 ± 1.94%	0.56 ± 0.67%	<0.001***
Inpatients	107	71	1.35 ± 3.31%	0.41 ± 0.65%	0.005**^#^
All patients	228	838	1.32 ± 2.67%	0.55 ± 0.67%	<0.001***

** *p* < 0.01; *** *p* < 0.001, ^#^ Welch correction, ^$^ excludes mixed-infection cases

To better understand the *P. falciparum* and *P. vivax* patient populations at GMC, univariate analyses using logistic regression with a primary outcome of malaria parasite species were performed. The demographic and basic diagnostic features associated with species of infection for all malaria-confirmed cases are shown in Table 2. Occupation as a construction worker was weakly correlated with *P. vivax* infection only (55%), albeit a significant proportion of *P. falciparum* cases (47%) self-identified as construction workers. Higher parasitaemia, lower Hb, anaemia, severe anaemia and hospitalisation were all significantly associated with *P. falciparum* infection. Conversely, gender, age, geographic origin, temperature, high fever, and severe fever did not significantly differ between *P. falciparum* and *P. vivax* cases.

**Table 2:** Univariate logistic regression of demographic and diagnostic features by species of malaria infection

Feature	*P. falciparum* ^$^	*P. vivax* ^$^	OR (95% CI)	*p* value
Male, n/N (%)	210/228 (92.1)	768/838 (91.6)	1.1 (0.6–1.9)	0.806
Age, y, Mean ± SD	27.0 ± 11.2	27.4 ± 10.5	1.0 (1.0–1.0)	0.659
Construction workers, n/N (%)	107/228 (46.9)	461/838 (55.0)	0.7 (0.6–1.0)	0.046*
Born in Goa, n/N (%)	30/228 (13.2)	96/838 (11.5)	1.2 (0.8–1.9)	0.337
Born in SW India, n/N (%)	56/228 (24.6)	187/838 (22.3)	1.1 (0.8–1.6)	0.475
Born in E India, n/N (%)	134/228 (58.8)	545/838 (65.0)	0.8 (0.6–1.1)	0.103
% Parasitaemia, Mean ± SD	1.3 ± 2.7	0.6 ± 0.7	1.6 (1.4–1.9)	<0.001***
Temperature, C, Mean ± SD	38.1 ± 1.6	38.2 ± 1.4	1.0 (0.9–1.0)	0.642
High fever (>38.1 °C), n/N (%)	104/228 (45.6)	380/838 (45.3)	1.0 (0.8–1.4)	0.769
Severe fever (>38.9 °C), n/N (%)	71/228 (31.1)	235/838 (28.0)	1.2 (0.9–1.7)	0.266
Haemoglobin, g/dL, Mean ± SD	10.9 ± 2.6	11.6 ± 2.1	0.9 (0.8–0.9)	<0.001***
Anaemia, n/N (%)	42/219 (19.2)	90/837 (10.8)	2.0 (1.3–2.9)	0.001**
Severe anaemia, n/N (%)	15/219 (6.8)	14/837 (1.7)	4.3 (2.0–9.2)	<0.001***
Hospitalisation, n/N (%)	107/228 (46.9)	71/838 (8.5)	9.1 (6.4–13.0)	<0.001***

* *p* < 0.05; ** *p* < 0.01; *** *p* < 0.001, ^$^ excludes mixed-infection cases

### Clinical characteristics of hospitalised confirmed malaria patients

Overall, 46.9% (107/228) of enroled *P. falciparum* patients were hospitalised compared with 8.5% (71/838) of enroled *P. vivax* patients, demonstrating an expected strong skew toward inpatient care and treatment of *P. falciparum* versus *P. vivax* (logistic regression; *p* < 0.001*)*. Furthermore, there was a significant age-dependent increase in hospitalisations. All malaria-positive patients over 40 years of age infected with either *P. falciparum* or *P. vivax* were significantly more likely to be hospitalised than those between the ages of one and 20 and ages 21 and 40 with the same species of infection (logistic regression; *P. vivax p* < 0.001, *P. falciparum p* = 0.033).

Among inpatients with malaria-confirmed infections at GMC, those with *P. falciparum* were more severely ill according to the WHO severe malaria criteria (Table 3) [42, 43]. Approximately equal percentages of hospitalised *P. falciparum* (76.6%) and *P. vivax* (78.9%) cases presented with at least one severity indicator. Of these criteria, respiratory distress, jaundice and renal failure were the most common symptoms present among both *P. falciparum* (51, 40.7, 42%) and *P. vivax* (42.9, 32.1, 19.7%). However, a significantly greater percentage of *P. falciparum* inpatients presented with at least two (43.9%, *p* < 0.05) and at least three (29.9%, *p* < 0.01) severity features compared with *P. vivax* inpatients, indicating that multi-organ involvement was much more common in hospitalised *P. falciparum* cases. There were six deaths among the 182 enroled malaria positive inpatients at GMC (6/182, 3.3%), all of which were *P. falciparum* cases (6/107, 5.6%).

**Table 3:** Univariate logistic regression of clinical features by species of malaria infection among hospitalised patients

Feature	*P. falciparum* ^$^	*P. vivax* ^$^	OR (95% CI)	*p* value
Glasgow coma score, Mean ± SD	13.8 ± 2.5	14.5 ± 1.5	0.8 (0.7–1.0)	0.040*
Coma, n/N (%)	10/107 (9.3)	2/71 (2.8)	3.7 (0.8– 17.9)	0.099
Blood urea nitrogen, mg %, Mean ± SD	75.7 ± 82.0	38.3 ± 24.7	1.0 (1.0–1.0)	0.003**
Serum creatinine, mg/dL, Mean ± SD	2.2 ± 3.2	1.1 ± 0.5	1.4 (1.1–2.0)	0.027*
Renal failure, n/N (%)	42/100 (42.0)	12/61 (19.7)	3.0 (1.4–6.3)	0.004**
Respiration rate (breaths/m), Mean ± SD	23.2 ± 6.7	21.8 ± 8.7	1.0 (1.0–1.1)	0.395
Respiratory distress, n/N (%)	50/98 (51.0)	27/63 (42.9)	1.5 (0.8–2.8)	0.219
Total bilirubin, mg/dL, Mean ± SD	5.9 ± 8.6	3.5 ± 4.1	1.0 (1.0–1.0)	0.812
Jaundice, n/N (%)	35/86 (40.7)	18/56 (32.1)	1.5 (0.8–3.1)	0.225
Severe jaundice, n/N (%)	15/86 (17.4)	5/56 (8.9)	2.3 (0.8–6.7)	0.136
Systolic BP, mm Hg, Mean ± SD	99.0 ± 13.0	95.8 ± 13.4	1.0 (1.0–1.0)	0.107
Shock, n/N (%)	3/105 (2.9)	5/70 (7.1)	0.4 (0.9–1.8)	0.217
Pulmonary oedema, n/N (%)	9/106 (8.5)	5/72 (6.9)	1.5 (0.8–3.1)	0.225
Abnormal bleeding, n/N (%)	7/107 (6.5)	1/71 (1.4)	5.0 (0.6–43.4)	0.136
% Parasitaemia, Mean ± SD	1.4 ± 3.3	0.4 ± 0.7	1.5 (1.0–2.1)	0.024*
Temperature, C, Mean ± SD	37.7 ± 1.5	37.5 ± 1.4	1.0 (0.9–1.2)	0.481
High fever (>38.1 °C), n/N (%)	35/107 (32.7)	22/71 (31.0)	1.2 (0.6–2.3)	0.599
Severe fever (>38.9 °C), n/N (%)	19/107 (17.8)	13/71 (18.3)	1.0 (0.5–2.3)	0.918
Haemoglobin, g/dL, Mean ± SD	10.1 ± 2.7	10.6 ± 2.5	0.9 (0.8–1.0)	0.159
Anaemia, n/N (%)	33/107 (30.8)	16/71 (22.5)	1.6 (0.8–3.3)	0.157
Severe anaemia, n/N (%)	12/107 (11.2)	2/71 (2.8)	4.6 (1.0–21.9)	0.049*
Days in hospital, Mean ± SD	6.0 ± 5.5	4.6 ± 2.0	1.1 (1.0–1.2)	0.044*
Long hospital stay (>8 days), n/n (%)	18/107 (16.8)	3/71 (4.2)	4.7 (1.3–17.2)	0.016*
Severity, Mean ± SD	2.0 ± 2.02	1.2 ± 1.14	1.3 (1.1–1.67)	0.005**
Severe malaria (SS > 0), n/N (%)	82/107 (76.6)	56/71 (78.9)	1.1 (0.5–2.2)	0.818
Severe malaria (SS > 1), n/N (%)	47/107 (43.9)	19/71 (26.8)	2.2 (1.2–4.3)	0.014*
Severe malaria (SS > 2), n/N (%)	32/107 (29.9)	9/71 (12.7)	3.0 (1.3–7.0)	0.007**

* *p* < 0.05; ** *p* < 0.01,^ $^ excludes mixed-infection cases, *SS* # of WHO severe malaria criteria met at admission and during hospitalisation

In addition to increasing clinical severity (*p* < 0.01), lower Glasgow coma score, higher blood urea nitrogen, higher serum creatinine, renal failure, per cent parasitaemia, severe anaemia, more days in the hospital and hospitalisation for more than eight days were all significantly correlated (*p* < 0.05) with *P. falciparum* infection. Shock or circulatory collapse was the only feature that showed a greater frequency among hospitalised *P. vivax* cases as compared to *P. falciparum*, albeit numbers were low in both species of infections. Though hypoglyemia and acidosis were not routinely measured, one case of hypoglycaemia was observed in a *P. falciparum* patient (1/45, 2.2%), while it was not seen among any of the tested *P. vivax* patients (0/13, 0%). Metabolic acidosis, when tested for, was observed more frequently in *P. falciparum* cases (12/33, 36.4%) as compared to *P. vivax* (2/19, 10.5%).

## Discussion

The present four year study was undertaken to better understand malaria disease burden in a peri-urban setting in India. The study points to a complex malaria situation in Goa with both *P. vivax* and *P. falciparum* cases year-round, a recent, substantial increase in the number of malaria-positive patients annually, a greater burden of disease from *P. falciparum*, and a highly diverse population of Indian patients from 26 of 29 states.

Between 2012 and 2015, GMC saw 6277 malaria-positive cases, with the number of *P. falciparum* and *P. vivax* patients increasing more than two-fold, from 889 in 2012 to 2261 in 2015. In contrast, between 2012 and 2015, the Indian National Vector Disease Control Programme (NVBDCP) reported 4854 cases of malaria in the entire state of Goa, 443 of which were caused by *P. falciparum,* and showed a more than two-fold decline in cases between 2012 (1714 cases) and 2015 (786 cases) [44]. Whether this discrepancy in malaria cases is due to an increase in the number of patients from other states, higher local transmission or some other cause remains to be determined. At the same time, these figures suggest that a large proportion of the total malaria cases in the state of Goa may be presenting to GMC and, thus, GMC may provide an approximation of malaria transmission and disease burden in the local community.

Despite the expected increase in number of malaria cases during and after the monsoon season, both *P. vivax* and *P. falciparum* patients were seen year-round at the GMC. While the year-round presence of *P. vivax* cases may be explained by the ability of *P. vivax* parasites to lie dormant as hypnozoites in liver stages with small numbers re-emerging during the dry season, this would not be the case for *P. falciparum* [8, 45]. Although it remains to be established, a more perennial transmission cycle of both *P. vivax* and *P. falciparum* may be aided by the recent discovery of a previously unsuspected vector in this region, *Anopheles subpictus* [46]. This vector has been shown to peak in numbers and transmission capacity both after the monsoons when the traditional urban malaria vector *Anopheles stephensi* peaks, but also in the dry season when *An. stephensi* numbers are low.

Although *P. vivax* infections were much more common at GMC, *P. falciparum* infections were associated with greater severity. Although approximately equal proportions of hospitalised patients with *P. falciparum* and *P. vivax* had at least one WHO severe malaria feature, patients with two or more severity criteria were more common to *P. falciparum* cases. The six deaths that occurred among study participants were all *P. falciparum* patients. In recent years, there have been a number of studies pointing to the importance of severe *P. vivax* in Asia, with some suggesting that *P. vivax* may be as or nearly as virulent as *P. falciparum* [47–52]. In the present study, though *P. vivax* caused some severe illness, multi-organ involvement was not typical. *P. falciparum* infections showed greater severity with substantial multi-organ involvement.

There was a strikingly greater proportion of adult males (86.7%) presenting to GMC with *P. falciparum* and *P. vivax* malaria than adult females. The same gender bias was smaller for children enroled in the study, with 64.7% male children and 35.3% female children. Such findings are consistent with previous reports of age-dependent sex-bias in clinical malarial disease in hypo-endemic regions [53] and with the gender distribution of malaria-positive patients at other MESA-ICEMR government tertiary hospital sites in India (unpublished data). It is also important to consider the profile of individuals who commonly seek care at government hospitals, which may be a contributing factor.

Both *P. falciparum* and *P. vivax* patients over 40 years of age were significantly more likely to be hospitalised by GMC than those between the ages of one and 20 years and 21 and 40 years, which points to a possible increase in the susceptibility of older populations to severe malaria in lower transmission settings. Future analysis will explore age-related symptomology and outcomes as well as parasite and gametocyte carriage to better understand if malaria virulence is increased in older populations in Goa. Other studies have shown that presenting syndromes in severe malaria depend on age and that age is an independent risk factor for a fatal outcome of malaria [16, 54, 55].

Construction sites in India are commonly suggested as potential transmission hot-spots, especially of severe *P. falciparum*, in low prevalence, relatively prosperous areas such as Goa [6, 56]. Though construction workers, who live and work at these sites and traditionally hail from the east and northeast states of India, accounted for roughly half of the malaria-positive study participants at GMC, they were nearly equally likely to be infected with *P. vivax* as *P. falciparum*. These findings may have implications for the conventional understanding of risk factors as well as the basis of targeted control measures at and around construction sites in the low transmission state of Goa. In the future, investigation to determine the possible effect of pre-existing *P. falciparum* and *P. vivax* immunity, compared across age, gender, origin and occupation, on transmission in Goa will be carried out.

There are a number of limitations to the present study. Due to logistical limitations and the high number of cases, only 18% of the hospital-identified malaria positive cases that presented to GMC over the study period were enroled. To date, there is not a complete, widely agreed-upon definition of what clinical features constitute severe *P. vivax* disease, as compared to severe *P. falciparum* disease, globally or on the Indian subcontinent [52, 57]. As such, the WHO definition of severe malaria for *P. falciparum* infection was used for both species, as is generally the case, which may bias or weaken associations seen among *P. vivax* cases. The present study does not include data for splenomegaly, spleen rupture or thrombocytopaenia, which have been reported to be common among severe *P. vivax* cases and are not reflected in the WHO criteria [22, 24, 52]. Lactate, hypoglycaemia, and metabolic acidosis, all of which are important indicators of severe malaria, were not routinely measured due to existing hospital practices. Malaria patients admitted to the GMC who required use of a ventilator, presumably due to respiratory distress [29, 47], which also has been commonly reported in severe *P. vivax,* were not included in the present study. This may impact the disease burden being reported among *P. vivax* and/or *P. falciparum* cases. It was also not always possible to accurately determine whether a patient had co-infections and/or had been treated with anti-malarials prior to presentation at GMC, a tertiary care centre. Co-infections and/or prior treatment may have impacted the measurement of clinical severity and/or course of malaria infection [58]. Future studies will seek to present the dynamic course of infection among severe malaria patients at GMC.

## Conclusion

The present study highlights the demographic, diagnostic and clinical profiles of *P. falciparum* and *P. vivax* patients at the only government tertiary care facility in a peri-urban setting in southwestern India. The number of malaria cases presenting to GMC increased more than two-fold from 2012 to 2015, which may be of interest in the context of elimination efforts. The data from GMC show a greater burden of disease contributed by *P. falciparum* than *P. vivax* and suggest a potential age-dependent increase in susceptibility that will be further investigated.

## Abbreviations

ACD: acid citrate dextrose
BP: blood pressure
BUN: blood urea nitrogen
DNA: deoxyribonucleic acid
DMID: Division of Microbiology and Infectious Diseases
GMC: Goa Medical College and Hospital
GOI: Government of India
Hb: haemoglobin
HCT: haematocrit
HMSC: Health Ministry Screening Committee
ICEMR: International Center of Excellence for Malaria Research
IMD: India Meteorological Department
IRB: Institutional Review Board
IQR: interquartile range
MESA: Malaria Evolution in South Asia
NIAID: National Institute of Allergy and Infectious Diseases
NIH: US National Institutes of Health
NVBDCP: National Vector Borne Diseases Control Programme
OR: odds ratio
RDT: rapid diagnostic test
SS: severity score
UW: University of Washington
WHO: World Health Organization
